# *Prickle1* regulates differentiation of frontal bone osteoblasts

**DOI:** 10.1038/s41598-018-36742-0

**Published:** 2018-12-21

**Authors:** Yong Wan, Brandi Lantz, Brian J. Cusack, Heather L. Szabo-Rogers

**Affiliations:** 1 Center for Craniofacial Regeneration, Department of Oral Biology, School of Dental Medicine, University of Pittsburgh, Pittsburgh, PA USA; 2 Department of Developmental Biology, School of Medicine, University of Pittsburgh, Pittsburgh, PA USA; 3 McGowan Institute of Regenerative Medicine, University of Pittsburgh, Pittsburgh, PA USA

## Abstract

Enlarged fontanelles and smaller frontal bones result in a mechanically compromised skull. Both phenotypes could develop from defective migration and differentiation of osteoblasts in the skull bone primordia. The Wnt/Planar cell polarity (Wnt/PCP) signaling pathway regulates cell migration and movement in other tissues and led us to test the role of *Prickle1*, a core component of the Wnt/PCP pathway, in the skull. For these studies, we used the missense allele of *Prickle1* named *Prickle1*^*Beetlejuice*^ *(Prickle1*^*Bj*^). The *Prickle1*^*Bj/Bj*^ mutants are microcephalic and develop enlarged fontanelles between insufficient frontal bones, while the parietal bones are normal. *Prickle1*^*Bj/Bj*^ mutants have several other craniofacial defects including a midline cleft lip, incompletely penetrant cleft palate, and decreased proximal-distal growth of the head. We observed decreased Wnt/β-catenin and Hedgehog signaling in the frontal bone condensations of the *Prickle1*^*Bj/Bj*^ mutants. Surprisingly, the smaller frontal bones do not result from defects in cell proliferation or death, but rather significantly delayed differentiation and decreased expression of migratory markers in the frontal bone osteoblast precursors. Our data suggests that *Prickle1* protein function contributes to both the migration and differentiation of osteoblast precursors in the frontal bone.

## Introduction

The craniofacial complex can be divided into three distinct regions: the cranial base, the skull vault and the face. The cranial base is the floor of the braincase and is composed of the presphenoid, basisphenoid, and basioccipital bones. The skull vault is composed of the frontal, parietal and interparietal bones providing the roof of the braincase. The bones of the cranial base are formed by endochondral ossification, while osteogenesis in the skull vault occurs by intramembranous ossification. Both the skull vault and cranial base have a composite embryological origin. In the mouse, the anterior regions (frontal, ethmoid, presphenoid, basisphenoid bones) are mostly neural crest-derived, while the posterior regions (parietal, basioccipital bones) are mesodermally derived^1–3^. In contrast, the facial skeleton is composed of intramembranous bones that are neural crest-derived^1^.

The development of the frontal bone begins with the migration of neural crest cells from the dorsal edge of the neural tube into the region anterior to the eye^2,3^. The cells settle there by E12.5 and express *Alkaline phosphatase (ALP)*, and *Runx2*. Subsequently, a sub-population of these cells migrate anteriorly to insert into the growing osteogenic front^3,4^ Heterozygote *Twist1* mouse mutants develop coronal synostosis^5,6^. The allelic series of *Msx2* and *Twist1* mutants suggests that these proteins interact cooperatively for osteoblast proliferation and differentiation in the frontal bone. The most severe phenotypes are observed in the *Msx2*^*−/−*^*; Twist1*^+/−^ compound mutants^6^. Decreased *Runx2* protein function causes cleidocranial dysplasia in both humans and mice^7–12^. The causes of craniosynostosis and frontal bone insufficiency could result from early defects in setting up the initial frontal bone condensation, or later problems with the migratory populations of cells. The mesenchymal deletion *(Dermo1-cre)* of *Wntless (Wls*) decreases the apical extension of the frontal bone^13^. The extension of the frontal bone relies on both β-catenin and *Twist1*^14^. The combined loss of all Wnt ligands in the *Wls* mutants and the ability of *Twist1* to direct cell movements in the absence of β-catenin imply that both Wnt/PCP and Wnt/β-catenin signaling may be required for apical extension of the frontal bone^13,14^.

The *Bj* mutants have a non-synonymous point mutation in *Prickle1* (C161F), a core component of the non-canonical Wnt/planar cell polarity (PCP) pathway^15–20^. *Prickle1* is a cytoplasmic protein with a single PET domain, three LIM domains and C-terminal Prickle homologous (PKH) domain. The *Bj* C161F mutation in *Prickle1* occurs in the first cysteine knot of the first LIM domain and is predicted to be deleterious to protein function^20^. *Prickle1* is required for epiblast apical-basal polarity and neuronal morphogenesis in mice^21,22^. Mutations in the human *Prickle1* are associated with familial epilepsy^23,24^. *Prickle1* mediates some of the WNT5A-associated genetic defects in Robinow syndrome^19^. The *Bj* mutant mice phenotype is consistent with another independent point mutation of *Prickle1*, C251X, including stunted limbs and cleft palate^20,25,26^.

*Prickle1*^*Bj/Bj*^ mutants develop cardiac outflow tract misalignment and cleft palate which both contribute to the perinatal death of the mutant mice^20^. *Prickle1* is widely expressed in the cytoplasm of the epiblast, as well as limb, but little is known about the role of Prickle1 in craniofacial osteogenesis. We hypothesize that the insufficient frontal bones result from the delayed differentiation and migration of osteoblast precursors cells.

## Results

### Craniofacial defects in the *Prickle1*^*Bj/Bj*^ fetuses

**Figure 1:**
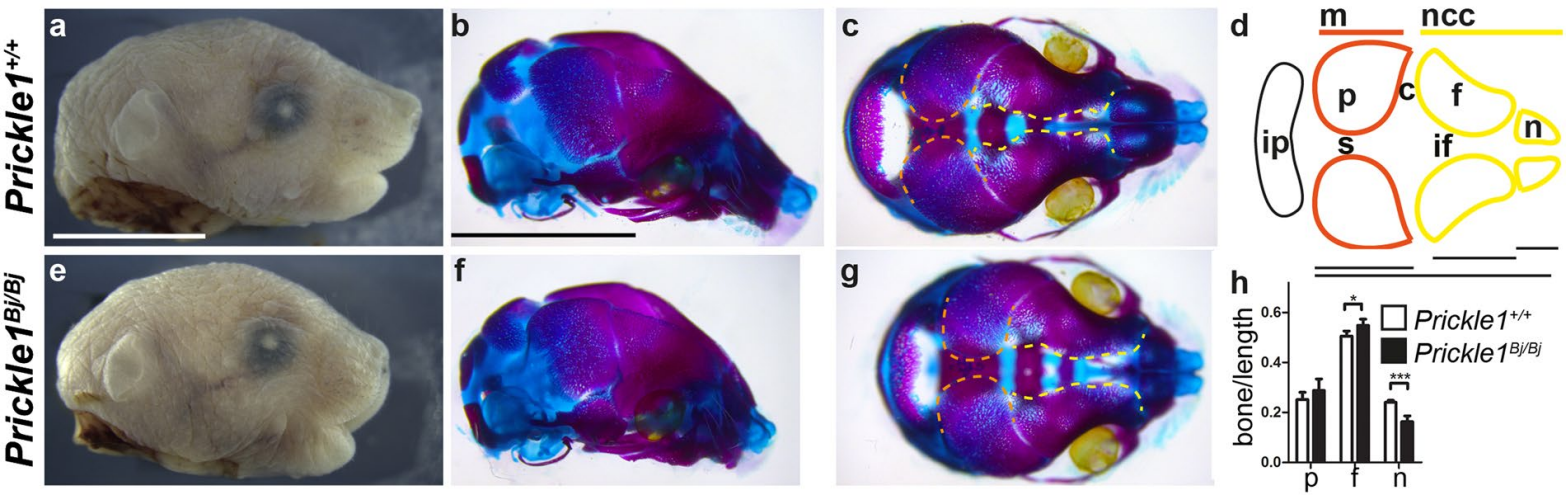
*Prickle1*^*Bj/Bj*^ mutants are microcephalic and have defects in the neural crest-derived skull. (**a**–**c**,**e**–**g**) Macroscopic views of *Prickle1*^+/+^ (**a**–**c**) and *Prickle1*^*Bj/Bj*^ (**e**–**g**) littermates. (**d**) Schematic of the superior view of the skull vault. (**h**) quantification of skull vault measurements. (**a,b,e,f**) The *Prickle1*^*Bj/Bj*^ mutant heads are shorter proximal-distally when observed laterally from the external (**e**) and alizarin red and alcian blue stained specimens (**f**). (**c,g**) Superior (birds-eye) view of the skull vault demonstrates that the interfrontal suture (yellow lines). (**d**) Schematic of the skull vault showing the tissue of origin and below is the schema for the measurements in (**h**). (**h**) The proximal-distal shortening of the *Prickle1*^*Bj/Bj*^ mutants is most profound in the nasal region. c, coronal suture; f, frontal bone; if, interfrontal suture; ip, interparietal bone; m, mesoderm; n, nasal bone; ncc, neural-crest cell; p, parietal bone; s, sagittal suture. Scale bar = 0.5 cm.

We have briefly described the microcephalic phenotype in the *Prickle1*^*Bj/Bj*^ mutants previously^20^. Here we present a detailed analysis of the skull vault. To begin to understand the underlying craniofacial phenotypes in the *Prickle1*^*Bj/Bj*^ mice, we performed an analysis of the morphology of the bones and cartilages of the head using alcian blue and alizarin red respectively. We noticed that the *Prickle1*^*Bj/Bj*^ skulls are smaller, and the proximal-distal length of the head is reduced with a concomitant increase in the medial-lateral width of the skull (Fig. 1b,c,f,g). The *Prickle1*^*Bj/Bj*^ viscerocranium, including the nasal, premaxillary, and maxillary bones, develops normal morphology, but these features are compressed proximally-distally (Fig. 1e,f,g). In the skull vault, we found that the interfrontal suture is enlarged in the *Prickle1*^*Bj/Bj*^ mutants while the sagittal suture is unaffected (Fig. 1g). We also observe that the mesodermally-derived skull is unaffected, including parietal, occipital and basioccipital bones (Fig. 1f,g). We measured the proximal-distal length of the frontal, parietal, and nasal bones and normalized them to the length of the skull vault. We found a statistically significant decrease in the contribution of the nasal bone to the total length of the skull vault. In contrast, the contribution of the frontal bone to the total length is increased, while the proportion of the parietal bone is unchanged in the *Prickle1*^*Bj/Bj*^ mutants (n = 3; Fig. 1h). Taken together, these results suggest that *Prickle1* protein function is required for all stages of frontal bone development.

**Figure 2:**
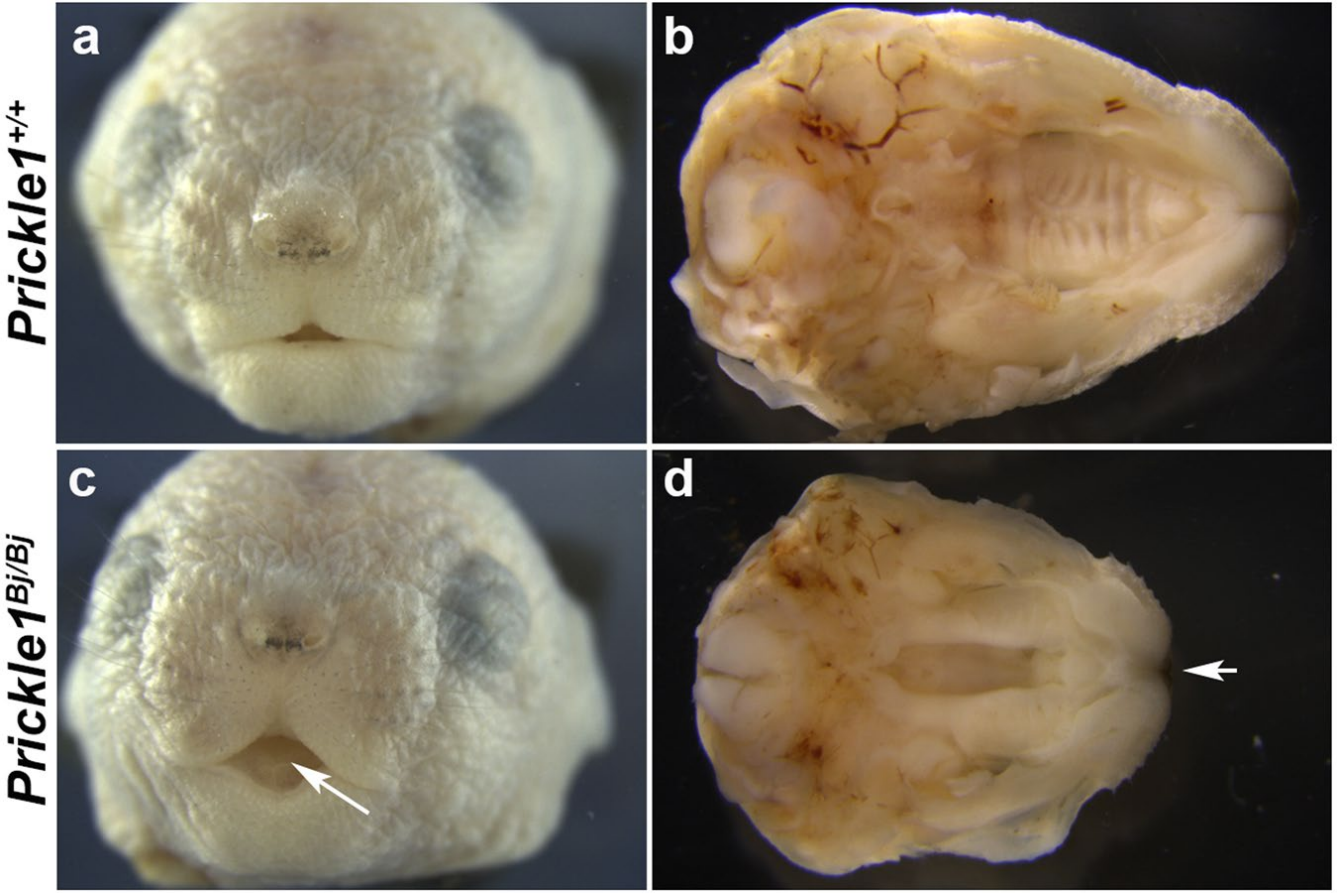
*Prickle1*^*Bj/Bj*^ mutants develop median cleft lip and cleft secondary palate. (**a,c**) Frontal views of wild-type (**a**) and *Prickle1*^*Bj/Bj*^ (**c**) at P0. Median cleft lip indicated by arrow and is completely penetrant. (**b,d**) Palatal view of E18.5 fetuses. Arrow indicates medial cleft lip and cleft secondary palate.

In addition to the microcephalic phenotype type (Figs 1e and 2c,d), we also observed several other craniofacial anomalies in the *Bj* mutants. All late stage fetuses developed a median cleft lip (Fig. 2c, n = 31/31). We observed a cleft palate between the palatine processes of the maxillary bones (Fig. 2d). The cleft palate is partially penetrant with 52% (n = 16/31) of *Prickle1*^*Bj/Bj*^ affected. As expected, with the cleft palate phenotype, the maxillary bones failed to meet in the midline (Fig. 2d). In agreement with Yang *et. al*.^25^, the cleft extends through the entire proximal-distal axis of the secondary palate (Fig. 2d).

### The frontal bone osteoblasts express Prickle1 and show decreased apical mineralization in *Prickle1*^*Bj/Bj*^

**Figure 3:**
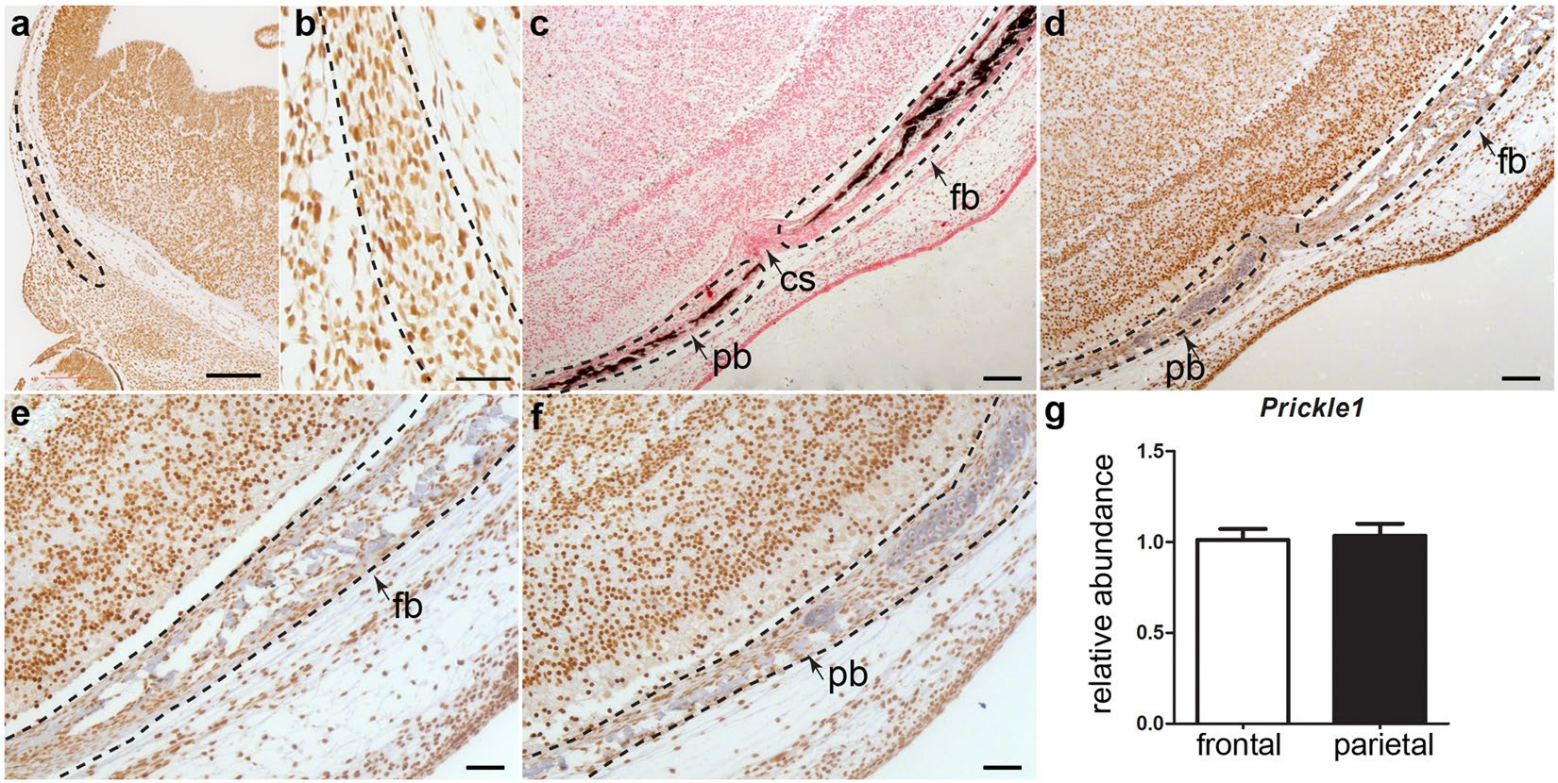
*Prickle1* protein is expressed in the craniofacial mesenchyme. We performed immunohistochemistry to *Prickle1* protein at E12.5 (**a,b**) and E15.5 (**d**–**f**) and detected *Prickle1* mRNA expression via Q-PCR at E15.5 (**g**). (**a**) Prickle1 protein is found in the frontal bone condensation at E12.5 (black outline). (**b**) A subset of the cells in the frontal bone express Prickle1 protein at E12.5 (black outline). (**c**) A horizontal section through the E15.5 skull showing von Kossa stained mineralized tissue in the frontal and parietal bones. (**d**) Section adjacent to c, stained with immunohistochemistry to Prickle1. (**e**) The E15.5 frontal bone expresses Prickle1 protein. (**f**) The E15.5 parietal bone also expresses Prickle1 protein. (**e**) At E15.5, *Prickle1* mRNA expression is equivalent between the frontal and parietal bones (n = 7). Scale bars: a = 100 μm; b-f = 200 μm. cs, coronal suture; fb, frontal bone; pb, parietal bone.

We have chosen to focus our analysis on the function of *Prickle1* in the developing skull vault and examined the tissue distribution of Prickle1 protein in the skull vault of wildtype embryos. At E12.5 we found that Prickle1 protein is expressed in a subpopulation of frontal bone osteoblasts (Fig. 3a,b). Later, Prickle1 protein is found in the frontal and parietal osteoblasts at E15.5 (Fig. 3c–f). We tested whether the frontal bones had a more severe phenotype because they differentially express *Prickle1* mRNA, however using qPCR, we observed that the frontal and parietal bones express similar level of *Prickle1* mRNA (Fig. 3g).

**Figure 4:**
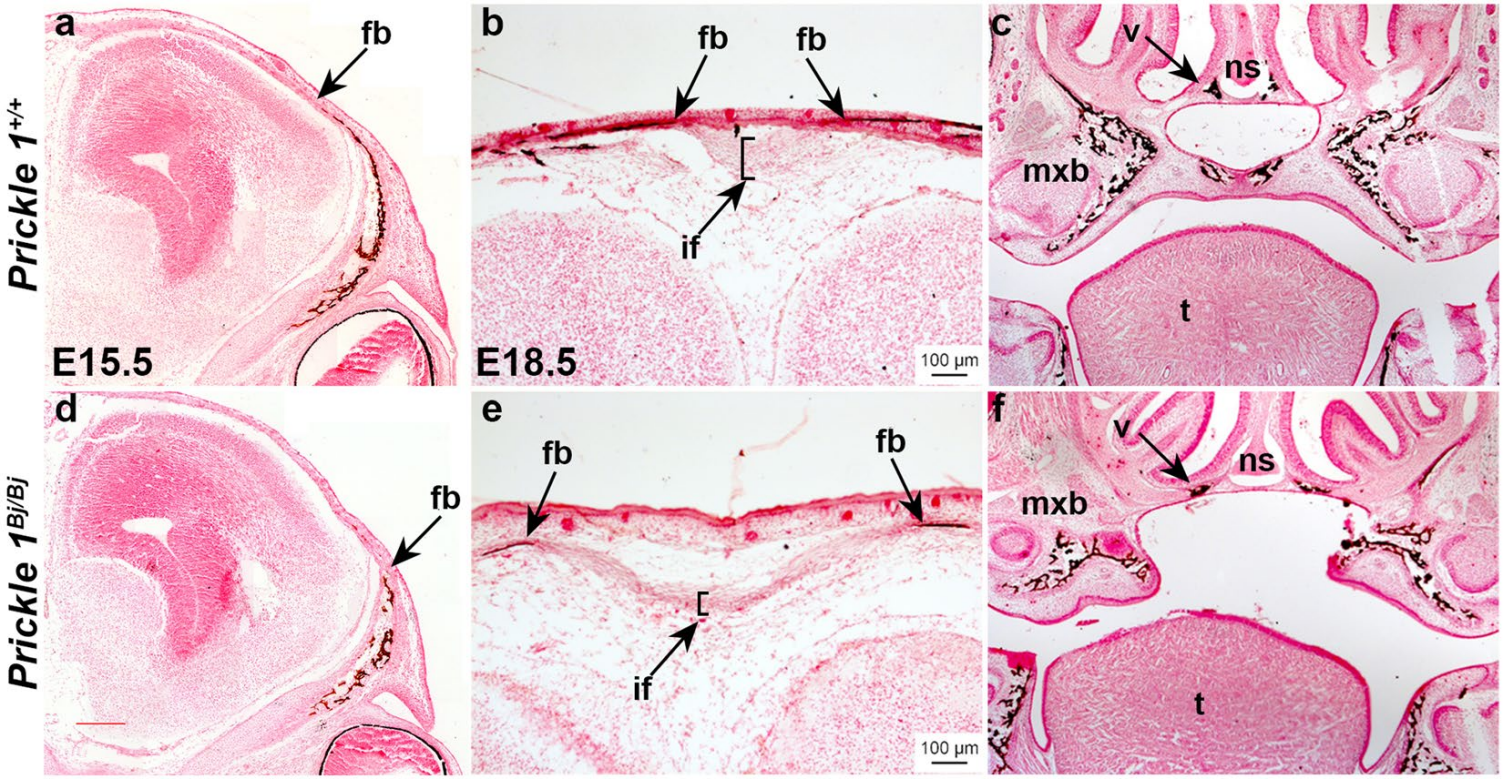
Mineralization defects in the *Prickle1*^*Bj/Bj*^ mutants. Histological analysis with von Kossa staining of *Prickle1*^+/+^ (**a**–**c**) and *Prickle1*^*Bj/Bj*^ (**d**–**f**) littermates at E15.5 (**a,d**) and E18.5 (**b,c,e,f**). (**a,c**) The smaller size of the *Prickle1*^*Bj/Bj*^ frontal bones is apparent at E15.5 and the osteogenic fronts do not extend as far apically as the littermate control (black arrow). (**b,e**) The interfrontal suture is enlarged between the *Prickle1*^*Bj/Bj*^ frontal bones (arrows). (**c,f**) The secondary palate is cleft and the maxillary and vomer (arrow) bones are displaced laterally. if, interfrontal suture; fb, frontal bone; mxb, maxillary bone; ns, nasal septum; t, tongue; v, vomer. Scale bar = 100 μm and applies to all.

We confirmed the wholemount skeletal findings by performing a histological analysis of the *Prickle1*^*Bj/Bj*^ and their littermate controls. We sectioned the samples coronally and stained them with Von Kossa to determine the mineralization status in these animals. We found that the craniofacial bones have begun mineralization by E15.5, which continues through E18.5 as evidenced by von Kossa staining (Fig. 4d–f). At E15.5, and E18.5, we found that the *Prickle1*^*Bj/Bj*^ frontal bones did not extend as far apically as their littermate controls (Fig. 4d–f). As expected from the alizarin stained specimens, we observed that the interfrontal suture is expanded mediolaterally with the loss of anterior-posterior growth of the sutural blastema (Fig. 4e, arrows, and bracket). In the nasal septum, the posterior regions of the vomer flare laterally away from each other (Fig. 4f).

### Proliferation and apoptosis are unaffected while differentiation is delayed in *Bj* mutants

**Figure 5:**
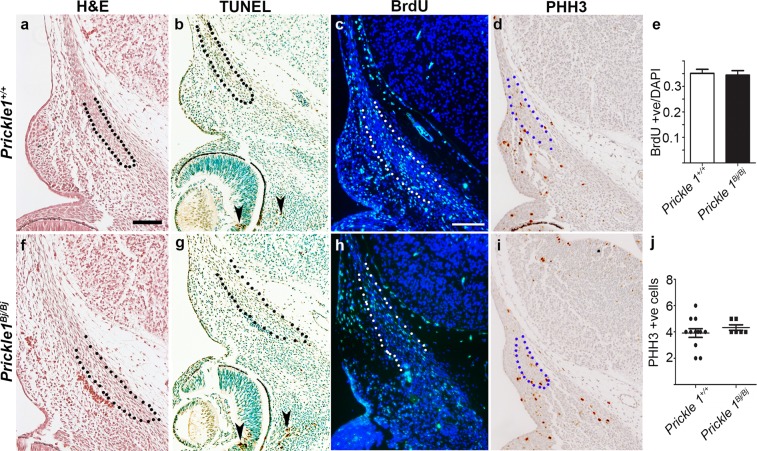
No change in the rate of proliferation or apoptosis in the E12.5 *Prickle1*^*Bj/Bj*^ frontal bones. E12.5 *Prickle1*^+/+^ (**a**–**d**) and *Prickle1*^*Bj/Bj*^ (**f**–**i**) littermates assayed for histology (haemotoxylin and eosin) staining (**a,f**), TUNEL staining (**b,g**), proliferation with BrdU immunofluorescence (**c,h**) and mitosis with phospho-histone H3 immunohistochemistry (**d,i**). (**a,d**) The frontal bone mesenchymal condensation (black outline) is present in both wildtype (**a)** and *Prickle1*^*Bj/Bj*^ (**f**) littermates. (**b,g**) TUNEL-positive cells were found near the eye (arrowheads) and absent in the frontal bone primordia. (**c,h**) BrdU-positive cells (green) are found in the frontal bone primordium (white outline) of wildtype (**c**) and *Prickle1*^*Bj/Bj*^ (**h**) littermates. (**d,i**) Few positive PHH3-positive cells (brown) are found in the frontal bone primordium of wildtype (**d**) and *Prickle1*^*Bj/Bj*^ (**i**) littermates. (**e**) No difference in the ratio of BrdU-positive cells in the frontal bone primordia (n = 3). (**j**) No difference in the number of PHH3-positive cells in the frontal bones between genotypes (n = 3). Scale bar = 100 μm.

Frontal bone insufficiency can result from defects in proliferation and cell death^27^. We undertook these studies at E12.5 because this is a critical stage for setting up the frontal bone condensation. Using H&E staining we observed the frontal bone condensation in wildtype and *Bj* frontal bone primordia (Fig. 5a,f). We found very few apoptotic (TUNEL-positive) cells in the frontal bone primordia in either genotype (Fig. 5b,g). We looked at the number of cells in S-phase using a BrdU pulse-chase labeling experiment. We counted the number of BrdU-positive cells and total number of nuclei, and found no difference in the ratio of proliferating cells between the *Bj* mutants and their littermate controls (Fig. 5c,h,e). We next tested the number of actively dividing cells using phospho-histone H3 immunohistochemistry. We found no differences in the number of dividing cells in the mutant frontal bone compared with the wild-type littermates (Fig. 5d,i,j).

**Figure 6:**
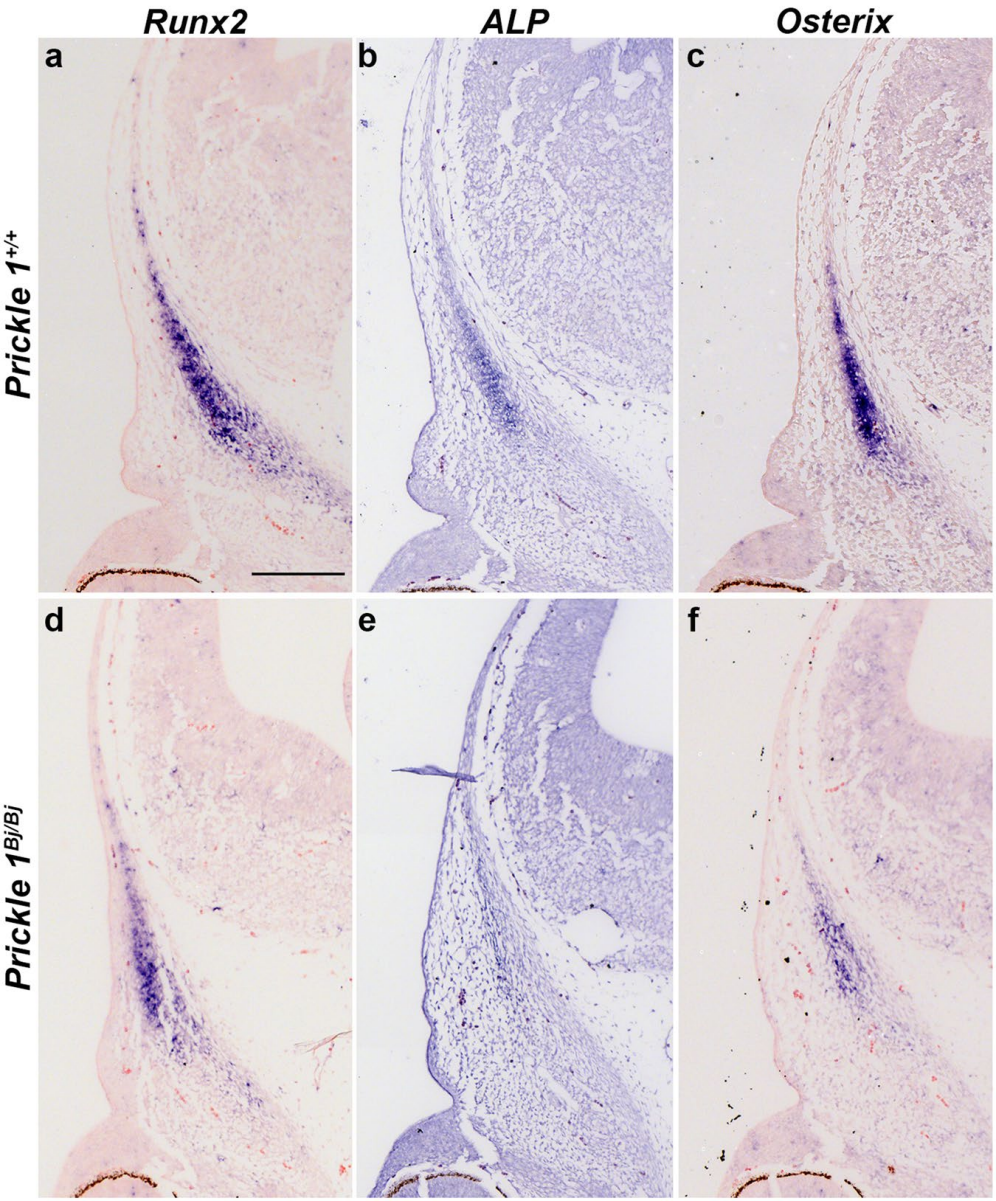
Ossification is delayed in the frontal bone primordium. DIG-labeled section *in situ* hybridization to E12.5 *Prickle1*^+/+^ (**a**–**c**) and *Prickle1*^*Bj/Bj*^ (**d**–**f**) littermates. The expression levels of *Runx2* (**a,d**), *Alkaline phosphatase* (*ALP*) (**b,e**) and *Osterix* (**c,f**) are decreased in frontal bone primordium of *Prickle1*^*Bj/Bj*^ mutants compared with wild-type control embryos. Scale bar in a = 200 μm, and applies to all.

Since we observed no changes in cell death or proliferation in the frontal bones, we determined whether osteogenic differentiation was occurring correctly. In the frontal bones, both *Alkaline phosphatase (ALP)* and *Osterix (Osx)* are expressed in pre-osteoblasts and osteoblasts. *Runx2* expression is an early marker of osteoblast commitment in the skull, while *ALP* is a marker of more mature osteoblasts. At E12.5 we observed that *Runx2* is expressed in the frontal bone primordium of both the *Prickle1*^*Bj/Bj*^ and littermate controls (Fig. 6a,d). However, the domain of expression in the *Prickle1*^*Bj/Bj*^ was much smaller, but the level of expression of *Runx2* was similar in both genotypes (Fig. 6a,d). The expression domains of *ALP* and *Osterix* were significantly smaller in size and signal intensity at E12.5 (Fig. 6e,f). At E15.5 we observed that the expression of both *Runx2*, *ALP* and *Osx* was decreased in the ectocranial layer of the mutant frontal bones (Supp Fig. 1) compared with the wild-type littermate. From this analysis we conclude that that intramembranous ossification is delayed in frontal bone.

### HH and Wnt signaling is decreased in the *Prickle1*^*Bj/Bj*^ frontal bones

**Figure 7:**
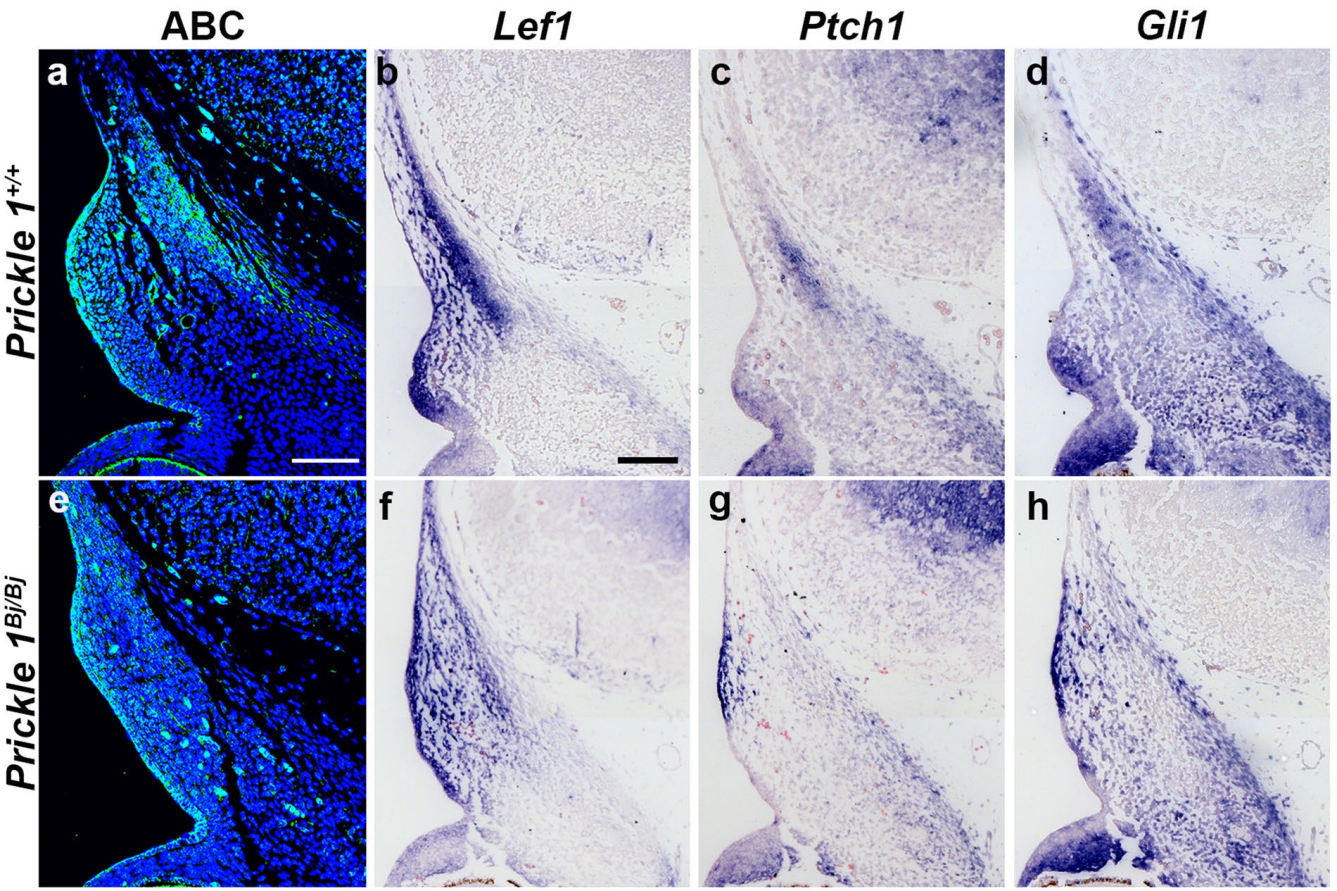
Wnt/β-catenin and Hedgehog signaling is decreased in the E12.5 frontal bone primordium. Immunofluorescence of active β-catenin (**a,e**) and DIG-labelled section *in situ* hybridization to *Lef1* (**b,f**), *Patched1 (Ptch1)* (c, g*)* and *Gli1* (**d,h**) in E12.5 *Prickle1*^+/+^ (**a**–**d**) and *Prickle1*^*Bj/Bj*^ (**e**–**h**) littermates. (**a,e**) The level of active β-catenin (ABC) protein is decreased in the frontal bone primordium, but is maintained in the surface epithelium in the *Prickle1*^*Bj/Bj*^ mutants compared with wild-type littermates. (**b,f**) *Lef1* expression is reduced in the *Prickle1*^*Bj/Bj*^ frontal bone primordium and throughout the mesenchyme surrounding it compared with wild-type littermates. (**c,g**) *Ptch1* expression is reduced in the *Prickle1*^*Bj/Bj*^ frontal bone primordium and throughout the mesenchyme surrounding it compared with control littermates. (**d,h**) *Gli1* expression is also reduced in the *Prickle1*^*Bj/Bj*^ frontal bone primordium compared with control littermates. Scale bar = 100 μm.

To determine if there is a defect in signaling that is leading to the delay in frontal bone osteogenesis, we sought to determine the level of canonical Wnt signaling and Hedgehog signaling in the frontal bones in the context of the *Prickle1*^*Bj*^ missense allele. As *Prickle1* is a negative regulator of Wnt/β-catenin signaling pathway^28^, we tested the level of active β-catenin in the frontal bone primordium and observed decreased active β-catenin in *Prickle1*^*Bj/Bj*^ mutant frontal bone primordia (Fig. 7a,e). In addition, we determined that downstream Wnt/β-catenin signaling is decreased as evidenced by the reduced *Lef1* expression in the *Prickle1*^*Bj/Bj*^ frontal bone (Fig. 7b,f).

The *Prickle1*^*Bj/Bj*^ animals showed defects in primary and motile cilia^20^ and several ciliopathy mutants have defects in frontal bone formation^29,30^. These defects suggest that the level of Hedgehog signaling could be defective in the *Prickle1*^*Bj/Bj*^ animals. In addition, HH signaling is required for the cranial bone development^31–33^. We tested the level of Hedgehog signaling via the expression of transcriptional targets of the pathway, *Ptch1* and *Gli1*. We found that the expression of both *Ptch1* and *Gli1* is decreased in the *Prickle1*^*Bj/Bj*^ mutants at E12.5 (Fig. 7g,h).

### Osteoblast migration is reduced in the *Prickle1*^*Bj/Bj*^ frontal bones

**Figure 8:**
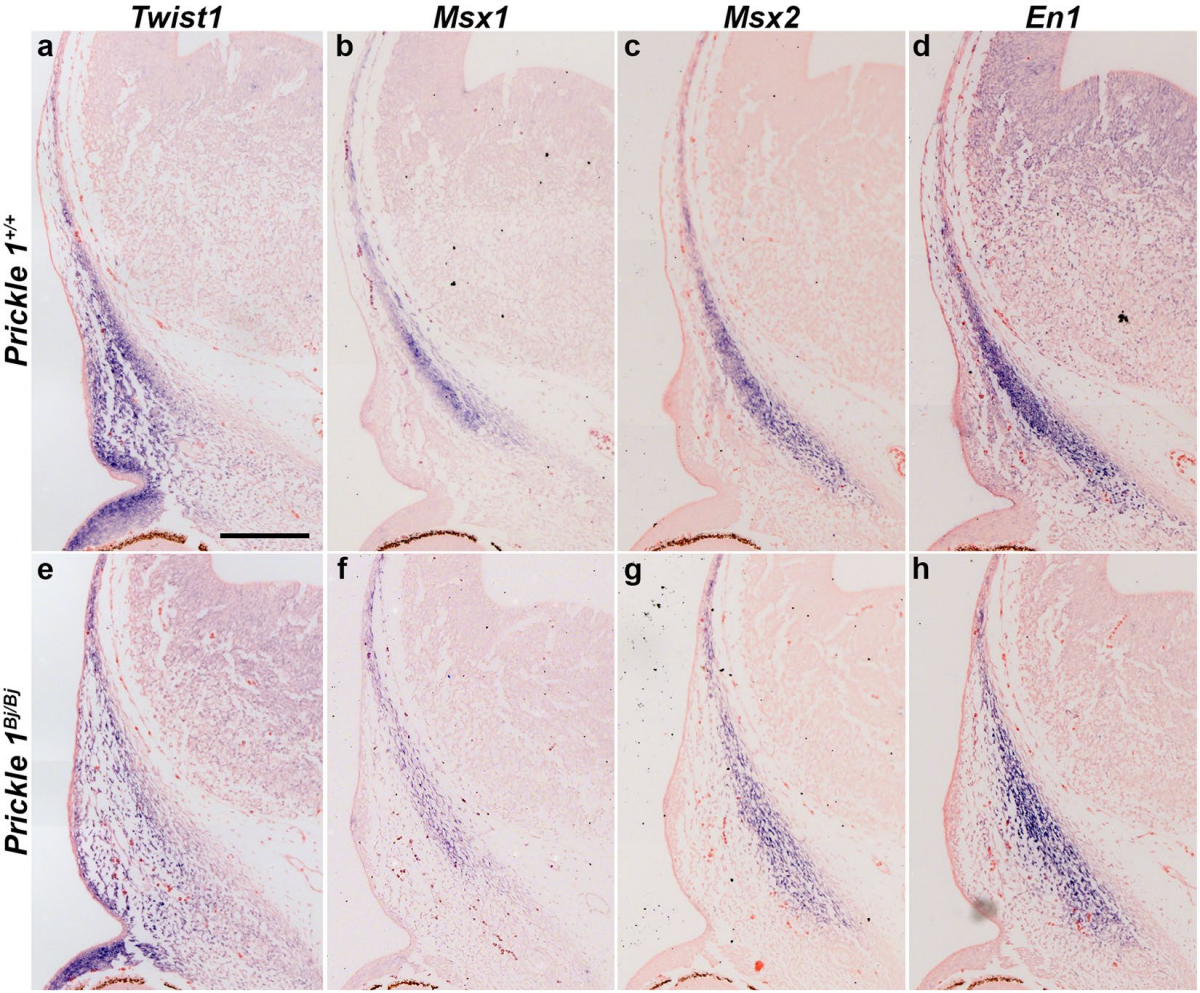
Osteoblast migration is decreased in the frontal bone primordium. DIG-labeled section *in situ* hybridization to *Twist1, Msx1, Msx2* and *Engrailed1 (En1)* to E12.5 *Prickle1*^+/+^ (**a**–**d**) and *Prickle1*^*Bj/Bj*^ (**e**–**h**) coronal sections. (**a,e**) Decreased expression of *Twist1* in the *Prickle1*^*Bj/Bj*^ compared with wild-type. (**b,f**) The expression of *Msx1* is decreased in the mutant compared with wild-type. (**c,g**) The expression of *Msx2* is slightly decreased in the *Prickle1*^*Bj/Bj*^ compared with wild-type. (**d,h**) The expression of *En1* is similar in the *Prickle1*^*Bj/Bj*^ compared with wild type. Scale bar in a = 200 μm, and applies to all.

The frontal bone develops in two parts: the body of the frontal bone differentiates *in situ* and mineralizes anterior to the eye, followed by apical extension towards the dorsal part of the skull by growth of the osteogenic front and insertion of migratory osteoblasts^3,4,34^. *Prickle1* protein is a core component in the Wnt/PCP pathway. One of the functions of the Wnt/PCP pathway is required for cell movement and intercalation. We wanted to test whether the *Prickle1*^*Bj/Bj*^ frontal bones were smaller because the *Prickle1* mutation decreases the migration potential of these cells. We performed whole mount *in situ* hybridization to markers of migratory populations of frontal bone cells *Twist1, Msx1, Msx2*, and *Engrailed1* (*En1*). We found decreased expression of *Twist1, Msx1, Msx2*, and *En1* in the frontal bone region by wholemount *in situ* staining (Supp Fig. 2a–d). We further confirmed this finding in coronal sections of E12.5 animals (Fig. 8). Indeed, we find that the expression of *Twist1, Msx1, Msx2* is decreased in the *Prickle1*^*Bj/Bj*^ frontal bone (Fig. 8e–g). The expression of *En1* is similar between genotypes in the frontal bone condensation, but is reduced in the dermis between the condensation and the surface ectoderm (Fig. 8h). These data support the hypothesis that the early apical migration of cranial mesenchyme is reduced in the mutant. During later stages, when the osteoblasts of the frontal bone are migrating to insert into the osteogenic front^4^, the expression of these three markers were examined in sagittal section of E15.5 wild type and mutant littermates. Our results show that the expression level of *En1*, *Twist1, Msx1* and *Msx2* in both the frontal bone and osteogenic front are reduced in the mutant (Supp Fig. 2i–l). These data suggest that *Prickle1* protein function is necessary for mediating cell migration of osteoblasts precursors during all stages of skull vault development.

## Discussion

We found that the *Prickle1*^*Bj*^ mutation results in defects in two processes in frontal bone development: delayed osteoblast differentiation and reduced migration in the frontal bone. The frontal bone defects that we observe are within the phenotypic spectrum for Cleidocranial Dysplasia (CCD). In humans, 70 percent of CCD cases result from mutations in *RUNX2* and have a triad of phenotypes including enlarged fontanelles, hypoplastic clavicles and supernumerary teeth. *In toto*, our *Prickle1*^*Bj/Bj*^ mutants look similar to the published allelic series that identified dose dependant and threshold requirements of *Runx2* in the development of the phenotypic spectrum of CCD^7,8^. Across the five different genotypes that express variable amounts of *Runx2*, the most consistent phenotype is the enlarged fontanelle and develops in concert with decreased *Osterix* expression^7,8^. In the *Prickle1*^*Bj/Bj*^ mice, we found both decreased *Runx2* and *Osx* expression in the frontal bone primordia, leading to smaller frontal bones and an enlarged fontanelle. These data suggest that Wnt/PCP signaling may be responsible for aspects of transcriptional control of *Runx2* expression. The link between mutations in *Prickle1*, Wnt/PCP signaling and the development of phenotypic variants in CCD patients warrants further exploration.

Bones develop through two different processes – intramembranous and endochondral ossification^35–37^. In the neural crest skeleton, the skull vault undergoes intramembranous ossification^1,38,39^. In mice, frontal bone development starts with mesenchymal condensations cranial to the eye at E12.5 and osteoblast differentiation begins at E13.5^36^. In *Beetlejuice* mutants, the frontal bone is hypoplastic. *Runx2*, *Osterix* and *ALP* expression domains are decreased at E12.5 and E15.5 with no observed changes in apoptosis or proliferation. We hypothesize that the domains are smaller because either fewer neural crest cells are recruited into the primordia or because the signaling environment surrounding the frontal bone primordia (including the dermis, dura and brain) inhibits the recruitment or maturation of the osteoblasts into the primordia. We are currently determining if the smaller frontal bones result from autonomous or non-autonomous functions of *Prickle1*.

The *Prickle1*^*Bj/Bj*^ skulls develop smaller but normally shaped frontal bones without affecting the parietal bones. We found that most of the proximal-distal shortening is found in the nasal bone, while the parietal bone is unaffected. Intriguingly, the *Prickle1*^*Bj/Bj*^ frontal bone contributes to a greater proportion of the proximal-distal length of the neurocranium. Molecularly, we observe decreased Wnt/β-catenin in the *Bj* mutant frontal bone primordium at E12.5. In agreement with previous reports regarding differences between the frontal and parietal bones^27,40^, we observe a defect only in the *Prickle1*^*Bj/Bj*^ frontal bone. Our data provides more support to the hypothesis that the parietal bone is not sensitive to changes in Wnt/β-catenin signaling and therefore develops normally in *Prickle1*^*Bj/Bj*^. We also observe decreased HH signaling in the *Bj* mutant frontal bone primordium at E12.5. The *Prickle1*^*Bj/Bj*^ frontal bone phenotype is consistent with the frontal bone phenotype observed in the *Ihh*^*-/-*^ mice^31,33^. Together the published phenotypes and our data suggest that both Wnt/β-catenin and HH signaling contribute to the migratory behavior of the frontal bones osteoblasts. We currently favor the model where the cells within the *Prickle1*^*Bj/Bj*^ mutant frontal bone primordia have prematurely sensed the completion of migration through *Prickle1* function. As a result, the osteoblasts decrease both Wnt and HH signaling, thus preventing additional migration.

*En1*, *Twist1* and *Msx1*, and *Msx2* are expressed during embryonic craniofacial bone development and are required for early migration and survival of cranial neural crest mesenchyme^6,41–45^. We found that *Twist1, Msx1*, and *Msx2* expression was reduced in the frontal bone primordia at E12.5 and E15.5 (Fig. 8, Supp Fig. 2). These results show that *Prickle1* is upstream of *Twist1* and *Msx2* to control apical expansion and migration of frontal bone osteoblasts. This suggests that there are subpopulations of neural crest cells that respond differently to the same signals–one population that is contributing to the suture and a second population that drives apical expansion of the frontal bone. However, from our analysis, we are unable to distinguish between whether the *Prickle1*^*Bj/Bj*^ osteoblasts have decreased migratory ability or whether there is a reduced population of apical migratory osteoblasts in the *Prickle1*^*Bj/Bj*^ frontal bone. Clarifying the identity, specific molecular markers and spatiotemporal location of these populations will be critical for understanding calvarial patterning and growth.

In this work, we have shown that the *Prickle1*^*Bj*^ mice are a new model for understanding the etiology of microcephaly^20^. The genetic, molecular and physical mechanisms underlying the development of microcephaly involves a combination of decreased growth of the craniofacial region and reduced expansion of the brain. Currently, few animal models exist to determine how growth patterns of the face and skull contribute to the development of microcephaly. We are currently undertaking work to determine how the migration of cells and expansion of each compartment (the brain, skull vault and cranial base) contributes to the development of microcephaly. We further hypothesize that decreased growth in all regions combined together contributes to the development of the microcephaly.

## Methods

### Mutant lines and animal husbandry

We obtained the founder animals from Dr. Cecilia W. Y. Lo (University of Pittsburgh) and maintained the *Beetlejuice* mutant line outbred on C57/BL6J background^20^. We genotyped the *Beetlejuice* line using a custom SNP assay (Invitrogen), Taqman Genotyping MasterMix, and read the results on a StepOnePlus machine. For embryo collection, we performed timed matings, and the day of the plug was designated as E0.5. For BrdU labeling, we intraperitoneally injected 10 mg/kg BrdU in sterile PBS one-hour prior to collection. The embryos were collected via C-section after CO2 euthanasia of the pregnant dam on the appropriate day and embryo staging was confirmed by morphology. Animal care and use described here was approved and complies with the guidelines of the Institutional Animal Care and Use Committee of the University of Pittsburgh.

### Skeletal preparation and histological analyses

After collection, embryos, and P0 fetuses were fixed in 4% paraformaldehyde at 4 °C overnight and embedded in paraffin using standard protocols. For histological analysis, 8-μm-thick sagittal and coronal sections were cut and placed on TESPA-coated Superfrost Plus slides. hematoxylin and eosin, Safranin O and Von Kossa staining were conducted using standard protocols. For wholemount staining of the skeleton, alcian blue and alizarin red staining, embryos of different stage as well as postnatal day (P0) mice were first fixed at 95% ethanol at room temperature overnight, and stained according to standard protocols^20^.

### Immunohistochemistry

#### Immunohistochemistry and immunofluorescence

Sections were blocked with 5% normal goat serum followed by primary antibodies incubation at 4 °C overnight. Antigen retrieval was conducted in sodium citrate buffer at 95 °C for 20 minutes. The primary antibodies used were as follows: Prickle1 (ab15577, Abcam) and active β-catenin (05–665, Millipore). We used the Alexa Fluor 488 conjugated goat anti-mouse IgG secondary antibody (A11001, Invitrogen). For immunohistochemistry with DAB detection, we used the primary antibodies above and the Vectastain ABC systems (Vector Labs) amplification system and ImmPACT DAB (Vector Labs) for detection.

#### Proliferation studies

BrdU samples underwent pre-treatment with Proteinase K, Exonuclease III and Dpn1. BrdU was detected using the RPN202 primary antibody (GE Healthcare Life Sciences) and Alexa Fluor 488 conjugated goat anti-mouse IgG secondary antibody. The samples were mounted with Prolong-Gold with DAPI (Invitrogen). The BrdU-positive cells and all cells (DAPI-stained) were counted in the frontal bone at E12.5 by two observers. The phospho-histone H3 (PHH3; #9701, CST) positive cells were detected using DAB. We compared the number of PHH3 positive cells and the ratio of BrdU-positive cells per frontal bone using a paired t-test (p < 0.05). Cells were counted in at least one section from three individual littermate pairs of wild-type and *Prickle1*^*Bj/Bj*^ mice.

### *In situ* hybridization

RNA *in situ* hybridization of both whole mount and section were performed according to standard protocols and detected with BM Purple^27^. The probes used were as follows: *Runx2, ALP, Osterix, En1, Twist1, Msx1, Msx2, Ptch1, Gli1*^27^. Each experiment was conducted in three different wild-type and mutant mice.

### Real-time PCR

RNA was extracted from frontal bone as well as parietal bone using the Trizol reagent (Invitrogen) according to standard procedures. Real-time PCR was performed on StepOnePlus Real-Time PCR System (Applied Biosystems) using a SYBR Green Kit (Roche). We performed ΔΔ-CT calculations with GAPDH as the control gene. Error bars represent the range for the expression for the seven samples and we compared expression using an unpaired T-test. The primers for Prickle1 are Forward: 5-TGCTCAGGAGATCCAAGTCC-3, Reverse: 5-CTCTCTTCAAAGTGATACGC-3.

### Microscopy, Imaging, and Measurement

Histological images were collected on a Zeiss Axioskop A1 microscope using a MrC3 camera and the Zen imaging software. Flourescent immunoflourescence images were captured on an Olympus Flou-View 100 confocal microscope. The images were compiled in Adobe Photoshop. Wholemount images were captured on a Leica M165FC dissecting microscope using a DFC450 camera with the Leica LAS software. We measured the proximal-distal length of the nasal, frontal and parietal bone as well as the total length of the skull vault using Leica Application Suite software (LAS v4.4). We used GraphPad Prism 5 to perform the Student’s t-test (p < 0.05).

The datasets generated during and/or analyzed during the current study are available from the corresponding author on request.

## Electronic supplementary material

Supplementary Figures
