# Supplementary material for: Prickle1 regulates differentiation of frontal bone osteoblasts
Source: Sci Rep. 2018 Dec 21;8:18021. doi: 10.1038/s41598-018-36742-0 (PMC6303328; doi:10.1038/s41598-018-36742-0)
Supplement: Supplementary file 1 — Supplementary Figures [file 41598_2018_36742_MOESM1_ESM.pdf]

Prickle1 regulates differentiation of frontal bone osteoblasts

Yong Wan[1], Brandi Lantz[1], Brian Cusack[1] and Heather L. Szabo-Rogers[1, 2, 3,]

[1]Center for Craniofacial Regeneration, Department of Oral Biology, School of Dental Medicine, University of Pittsburgh, Pittsburgh PA, 15213.

[2]Department of Developmental Biology, School of Medicine, University of Pittsburgh, Pittsburgh PA, 15213.

[3]McGowan Institute of Regenerative Medicine, University of Pittsburgh, Pittsburgh PA, 15213.

* Corresponding author

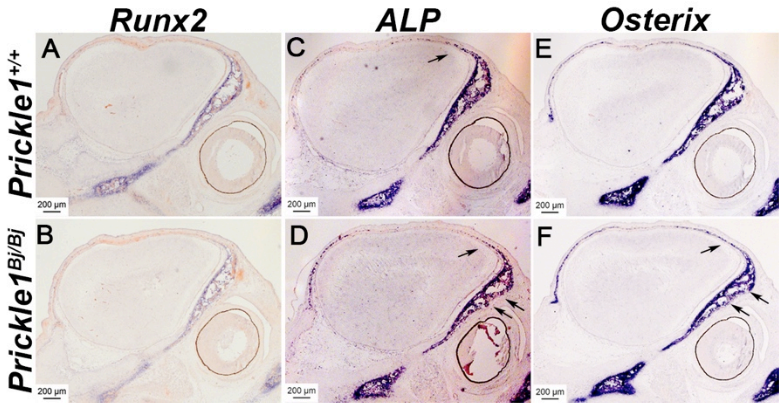

Supplementary Fig 1: Ectocranial differentiation is delayed at E15.5 DIG-labeled section in situ hybridization to E15.5 *Prickle1*$^{+/+}$ (a, c, e) and *Prickle1*$^{Bj/Bj}$ (b, d, f) littermates, (a, b) *Runx2* is expressed at similar levels in the mutant and wildtype frontal bones. (c, d) *Alkaline phosphatase* (*ALP*) expression is slightly decreased in ectocranial layer of the *Prickle1*$^{Bj/Bj}$ frontal bone (arrows). (E, F) *Osterix* expression is decreased in ectocranial layer *Prickle1*$^{Bj/Bj}$ frontal bone (arrows).

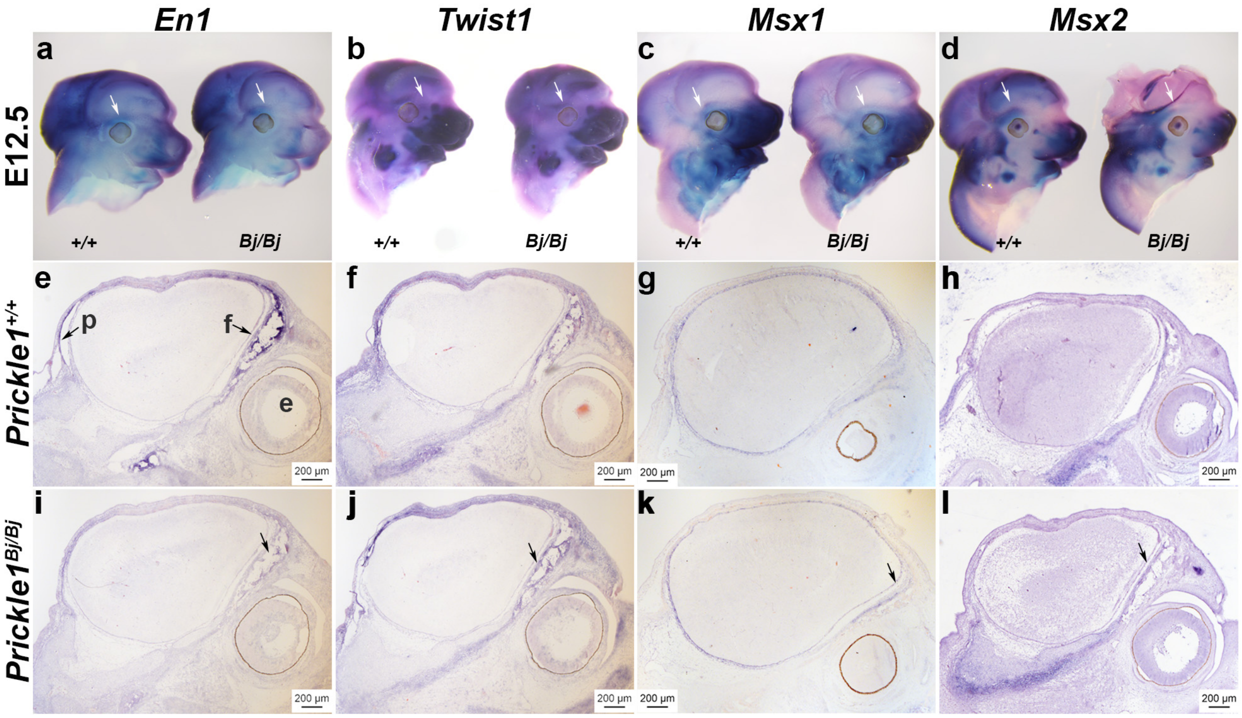

Supplementary Figure 2: Osteoblast migration is decreased in the frontal bones. DIG-labeled wholemount and section *in situ* hybridization to markers of osteoblast migration (*En1, Twist1, Msx1,* and *Msx2)* at E12.5 (a-d) and E15.5 (e-l) using *Prickle1*$^{+/+}$ and *Prickle1*$^{Bj/Bj}$ littermates. The supraorbital domain of *En1* expression is similar at E12.5, while the supraorbital domains of *Twist1, Msx1,* and *Msx2*, are reduced at E12.5 (white arrows). (e, i) Expression of *En1* is decreased at E15.5 in the *Bj* mutant. (f, j) *Twist1* expression is decreased. (g, k) *Msx1* expression is decreased. (h, l) *Msx2* expression is also reduced. +/+, *Prickle1*$^{+/+}$; *Bj/Bj, Prickle1*$^{Bj/Bj}$; f, frontal; p, parietal.
